# Clinical and Radiographic Outcomes of Electrolytic Implant Surface Decontamination in Peri-Implantitis Surgery: A Retrospective Cohort Study

**DOI:** 10.3390/dj14070447

**Published:** 2026-07-16

**Authors:** Nicola De Angelis, Paolo Pesce, Giulia Santamaria, Esteban Colombo, Catherine Yumang, Maria Menini

**Affiliations:** 1 Department of Surgical Sciences and Integrated Diagnostics (DISC), University of Genoa, 16126 Genova, Italy; 2 Private Practice, 15011 Acqui Terme, Italy

**Keywords:** peri-implantitis, dental implants, biofilms, electrolysis, osseointegration, oral surgical procedures

## Abstract

**Background:** To compare the short-term clinical and radiographic outcomes of electrolytic versus conventional mechanical implant surface decontamination during surgical treatment of peri-implantitis. **Methods:** This retrospective cohort study included 20 implants affected by peri-implantitis in 20 patients. Ten implants were treated using electrolytic implant surface decontamination, while 10 implants received conventional mechanical decontamination with titanium curettes, chitosan brushes, and glycine powder air-polishing. In addition, regenerative therapy of the peri-implant defect was performed in 8 of the 20 treated sites, equally distributed between the two groups (4 sites per group). Clinical and radiographic parameters, including probing depth (PD) and radiographic bone loss, were assessed at baseline and after 6 months. Intragroup and intergroup comparisons were performed using parametric statistical analyses (α = 0.05). **Results:** Both treatment protocols resulted in statistically significant improvements in probing depth reduction and radiographic bone fill after 6 months (*p* < 0.001). In the electrolytic group, mean PD decreased from 6.7 ± 0.95 mm to 4.0 ± 0.2 mm, while in the conventional group, PD decreased from 6.8 ± 1.03 mm to 3.3 ± 0.3 mm. Radiographic bone loss decreased from 4.88 ± 0.70 mm to 0.94 ± 0.70 mm in the electrolytic group and from 4.88 ± 1.83 mm to 0.94 ± 1.14 mm in the conventional group. No statistically significant intergroup differences were observed for probing depth reduction or radiographic bone level changes. No implant losses occurred during the observation period. **Conclusions:** Within the limitations of this retrospective study, both electrolytic and conventional mechanical implant surface decontamination protocols resulted in significant short-term clinical and radiographic improvements following peri-implantitis surgery. Electrolytic cleaning demonstrated outcomes comparable to conventional therapy and may represent a viable alternative for implant surface decontamination.

## 1. Introduction

Dental implants are widely used for the rehabilitation of partially or fully edentulous patients and currently represent a predictable therapeutic option in contemporary dentistry. Titanium implants are among the most commonly used devices because of their favorable mechanical properties, biocompatibility, and ability to establish direct bone-to-implant contact through osseointegration [1,2]. The long-term stability of this interface is influenced by several factors, including implant material and design, bone quality, loading conditions, and surface characteristics [1,2].

Despite the high survival rates reported for dental implants, biological complications may occur after functional loading. Among these, peri-implantitis represents one of the most clinically relevant causes of late implant failure. The diagnostic framework of peri-implant diseases has progressively evolved over the last decades. The term peri-implantitis, introduced by Mombelli in 1987 [3], was initially used to describe an inflammatory pathological process affecting the tissues surrounding osseointegrated implants. Subsequently, the 2017 World Workshop, jointly promoted by the European Federation of Periodontology and the American Academy of Periodontology, defined more uniform diagnostic criteria, distinguishing peri-implant health, peri-implant mucositis, and peri-implantitis on the basis of clinical and radiographic parameters [4]. According to this classification, peri-implantitis is characterized by inflammation of the peri-implant soft tissues associated with progressive loss of supporting bone [4].

Although this classification has contributed to greater diagnostic standardization, further proposals have sought to improve its clinical applicability by integrating aspects such as loss of osseous support and the three-dimensional position of the implant [5].

The therapeutic management of peri-implantitis includes both non-surgical and surgical strategies. Non-surgical procedures generally represent the first therapeutic approach and aim to reduce the bacterial load through debridement and implant surface decontamination without surgical access. However, in more advanced cases, the clinical outcomes achieved with these methods may be only partial. This limitation may be related to the difficulty of obtaining complete decontamination of exposed implant surfaces, the complexity of implant morphology, and the persistence of bacterial biofilms that are difficult to control exclusively through conservative procedures [6,7].

In the presence of more advanced lesions or lesions that do not respond to initial therapy, surgical treatment represents a relevant therapeutic option and is often more appropriate than a non-surgical approach alone [8,9,10,11]. Flap elevation allows for direct visualization of the defect, more effective removal of inflammatory tissues, and more accurate decontamination of the implant surface, thereby contributing to the creation of more favorable biological conditions for healing.

Within surgical therapy, the available approaches can mainly be divided into two categories: non-augmentative and augmentative procedures [7]. Non-augmentative procedures are primarily aimed at controlling infection, reducing peri-implant pockets, and creating a tissue morphology that is more favorable for long-term hygiene maintenance. Augmentative procedures, on the other hand, aim to reconstruct the peri-implant bone defect through regenerative techniques, when defect morphology and clinical conditions allow it [8,9].

However, the literature has not yet identified a universally superior surgical protocol, and outcome predictability remains variable among the different therapeutic strategies available [12,13,14,15,16]. In this context, implant surface decontamination represents a crucial step in peri-implantitis surgery since the control of biofilms and contaminants present on the exposed surface may influence the resolution of inflammation and, in selected cases, regenerative healing.

Conventional approaches include several mechanical and chemical methods which may present limitations related to the difficulty of accessing implant threads and micro-rough surfaces, the possible incomplete removal of biofilms, and, in some cases, the risk of altering the characteristics of the implant surface [15,16]. In this scenario, electrochemical and electrolytic methods have been proposed as alternative strategies to promote biofilm removal from titanium implant surfaces while preserving surface microtopography and physical properties [17].

In addition to conventional mechanical and chemical decontamination approaches, laser-assisted implant surface decontamination has also been proposed as a potential adjunctive strategy in the treatment of peri-implantitis [18]. Among laser systems, the Er:YAG laser has attracted particular interest because of its ability to effectively remove biofilms and contaminated deposits from titanium implant surfaces while minimizing thermal damage when used with appropriate parameters. Clinical studies and systematic reviews have suggested that Er:YAG-assisted decontamination may improve peri-implant clinical parameters, including probing depth reduction and bleeding control, although the available evidence remains heterogeneous and does not currently support the clear superiority of one decontamination protocol over another [19]. Therefore, implant surface decontamination in peri-implantitis surgery remains an area of ongoing investigation, with no universally accepted gold-standard approach.

Electrolytic cleaning therefore represents an innovative approach for the decontamination of dental implants. In the GalvoSurge^®^ system, as described in the reference literature, the procedure involves the use of sodium formate as an electrolytic solution [15,17,20]. During the procedure, titanium implants are subjected to a controlled electrical current of up to 600 mA, while the electrolyte solution is delivered through a platinized ring. This process has been associated with the removal of carbonaceous contaminants from the implant surface, resulting in improved surface bioactivity and conversion toward a more hydrophilic state [20,21,22].

This aspect is clinically relevant, since hydrophilic titanium surfaces have been associated with improved blood clot stabilization, a more favorable cellular response, and enhanced angiogenesis during the early phases of osseointegration, thereby creating conditions that may be more favorable for bone formation [23,24,25]. These mechanisms may support regenerative procedures and contribute, in selected cases, to the possible re-osseointegration of implants [17].

Therefore, the aim of this retrospective cohort study was to compare the clinical and radiographic outcomes of two different implant surface decontamination protocols used during the surgical treatment of peri-implantitis. In cases where defect morphology was considered suitable for regenerative therapy, bone grafting was performed according to the same clinical criteria in both groups.

## 2. Materials and Methods

### 2.1. Sample Size Calculation and Power Analysis

Due to the retrospective design of the present study, no a priori sample size calculation was performed. Instead, a post hoc power analysis was conducted to estimate the statistical power associated with the available sample.

The study included a total of 20 implants (10 per group). The sample size was consistent with previous clinical investigations evaluating electrolytic implant surface decontamination. In particular, randomized controlled trials assessing electrolytic cleaning in peri-implantitis treatment reported a sample size of 12 implants per group, based on a power calculation using a significance level (α) of 0.05 and a statistical power of 90% [26]. These studies used G*Power software version 3.1.9.7 and assumed differences in clinical parameters such as probing depth and bone level changes as primary outcomes.

Similarly, other peri-implantitis intervention studies have considered a probing depth reduction of approximately 1 mm as clinically relevant, with an assumed standard deviation of about 1 mm for implant-related measurements [27].

Based on these assumptions, a post hoc power analysis was performed for the primary outcome (change in probing depth at 6 months), assuming:

- A clinically relevant difference (Δ) = 1.0 mm;
- Standard deviation (σ) = 1.0 mm;
- Two-sided α = 0.05;
- Equal allocation ratio (1:1).

Under these conditions, a total sample size of 20 implants (10 per group) was expected to provide adequate power, depending on the exact variance observed in the dataset. Based on these assumptions, the available sample size was considered sufficient for exploratory analyses and consistent with previous pilot investigations in this field.

However, the results should be interpreted with caution, and larger prospective trials are warranted to confirm the findings.

### 2.2. Study Design

This retrospective cohort study aimed to compare the clinical outcomes of two different implant surface decontamination protocols used during surgical treatment of peri-implantitis. Patients treated between January 2024 and December 2024 were retrospectively screened for eligibility. The study protocol was conducted in accordance with the Declaration of Helsinki and the STROBE guidelines, and was approved by the institutional Ethics Committee (CERA N. 2026/31).

### 2.3. Case Definition and Criteria

Peri-implantitis was defined according to the 2017 World Workshop on the Classification of Periodontal and Peri-Implant Diseases and Conditions [28] as the presence of bleeding and/or suppuration on probing, increased probing depth compared to previous records, and radiographic evidence of progressive bone loss beyond initial remodeling. In the absence of baseline data, a diagnosis was established when probing depth (PD) ≥ 6 mm, bone levels ≥ 3 mm apical to the most coronal portion of the intraosseous implant, and bleeding and/or suppuration on probing were present. The statistical unit was the patient. Only one implant per patient was included in the analysis; in cases presenting multiple affected implants, the implant showing the worst clinical condition was selected. Inclusion criteria were: ≥ 18 years of age; presence of at least one titanium dental implant diagnosed with peri-implantitis; indication for surgical intervention after unsuccessful non-surgical therapy; complete clinical and radiographic records at baseline and follow-up; minimum follow-up of 6 months; implants in use for at least 12 months. Exclusion criteria included: uncontrolled systemic conditions; history of head and neck radiotherapy; pregnancy or lactation; medications affecting bone metabolism; untreated or uncontrolled periodontitis; poor oral hygiene compliance (plaque index > 25%); heavy smoking (>10 cigarettes/day); implant mobility; incomplete records; previous surgical peri-implantitis treatment at the same site.

Implants were allocated into two groups based on the decontamination protocol performed:

- **Conventional mechanical decontamination (Control group):** titanium curettes, chitosan-based brushes, and glycine powder air-polishing.
- **Electrolytic decontamination (Test group):** implant surface cleaning using an electrolytic system.

### 2.4. Surgical Procedure

All interventions were performed under local anesthesia by experienced clinicians.

**Figure 1:**
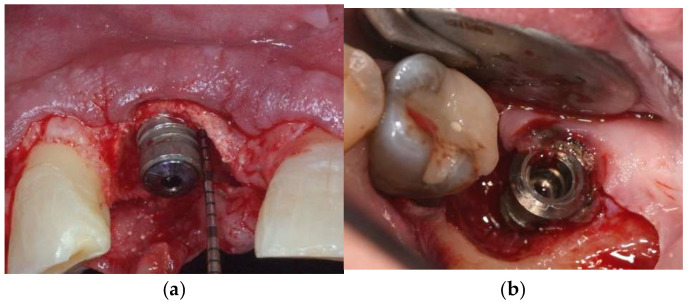
(**a**,**b**) Two cases after the debridement with evidence of exposed threads.

A crestal incision with or without releasing incisions was performed, and a full-thickness mucoperiosteal flap was elevated to allow for adequate visualization of the peri-implant defect. Granulation tissue was meticulously removed using titanium curettes to fully expose the contaminated implant surface (Figure 1).

#### 2.4.1. Implant Surface Decontamination

Before surgical decontamination, the prosthetic suprastructure/component was removed in all cases, regardless of treatment group, to facilitate direct access to the contaminated implant surface and peri-implant defect. During surgery, the peri-implant defect was evaluated clinically according to its morphology and configuration, and the indication for regenerative treatment was based on the operator’s intraoperative assessment of defect containment and suitability for grafting. Due to the retrospective nature of the present study, it is assumed that the decision on the surface decontamination system was related to the operator choice. This was considered a potential source of selection bias.

**Figure 2:**
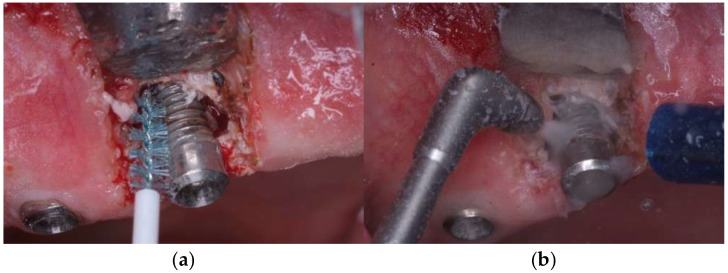
Control Group: conventional polishing of the exposed implant surface using (**a**) chitosan brushes; (**b**) glycine powder spray.

In the control group, implant decontamination was achieved through a combined mechanical approach including titanium curettes, chitosan-based brushes, and air-polishing with glycine powder (Figure 2).

**Figure 3:**
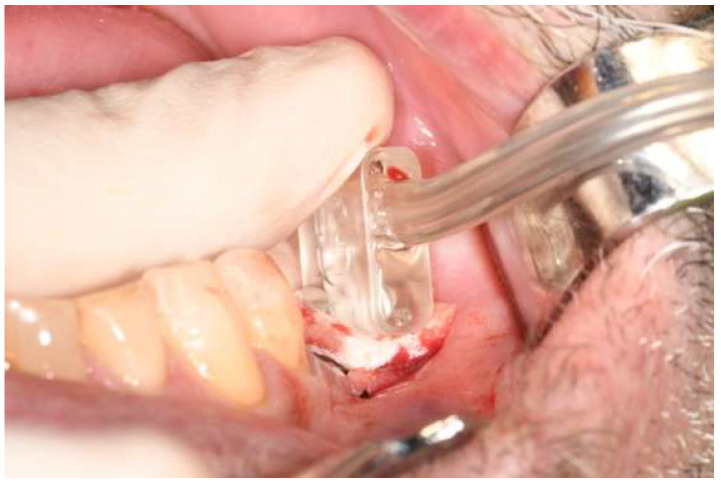
The disposable tube connected to the pin is inserted into the implant to allow the flow of the solution.

In the test group, implant decontamination was performed using an electrolytic cleaning system [17]. In this group, implant surface decontamination was performed exclusively using the electrolytic cleaning system, according to the manufacturer’s protocol. No adjunctive mechanical instrumentation of the implant surface was performed in this group. In contrast, implants allocated to the conventional group underwent surface decontamination by means of mechanical instrumentation according to the study protocol. This method is based on the application of a low-voltage electrical current in the presence of an electrolyte solution (typically sodium), delivered through a dedicated device. During the procedure, the implant surface acts as a cathode, while an external electrode completes the circuit. The electrochemical reaction results in the formation of hydrogen bubbles at the implant surface, which mechanically disrupt and detach the biofilm without altering the implant microtopography (Figure 3).

The electrolytic cleaning was applied circumferentially to the exposed implant surface following the manufacturer’s protocol, ensuring complete coverage of the contaminated area.

#### 2.4.2. Defect Management

Following decontamination, the morphology and depth of the peri-implant defect were assessed.

When the peri-implant defect presented an intraosseous component exceeding 3 mm and a morphology considered favorable for containment, regenerative therapy was performed according to the operator’s clinical judgment. The rationale for grafting was to provide structural support for blood clot stabilization and to facilitate defect management in contained or partially contained defects, in line with routine regenerative surgical principles. The defect was filled with a synthetic xenograft bone substitute, covered with native collagen porcine resorbable membrane. This threshold is supported by previous evidence indicating that deeper intraosseous defects are more amenable to regenerative procedures and may benefit from augmentation strategies [7,29,30].

**Figure 4:**
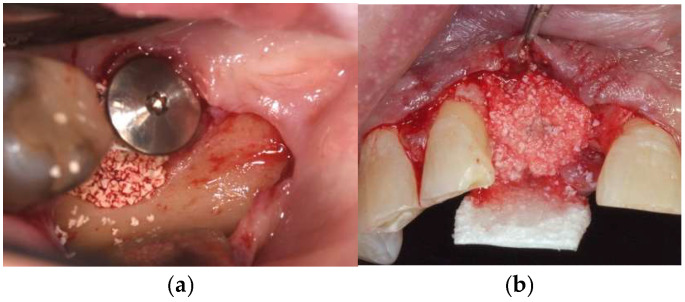
Regardless the decontamination treatment, peri-implant defects were grafted with synthetic bone graft following the above decision process. (**a**) Case from test group; (**b**) control group case.

For defects ≤ 3 mm, no regenerative procedure was performed, and a non-augmentative approach was adopted (Figure 4).

The flap was repositioned at its original level and sutured to achieve primary closure. Interrupted and/or mattress sutures were used to ensure tension-free adaptation of the soft tissues.

All patients received a standardized postoperative regimen:

- **Antibiotic prophylaxis:**
  Amoxicillin 2 g administered 1 h before surgery; in case of allergy, azithromycin 500 mg once daily for 3 days starting the day before surgery.
- **Antiseptic therapy:**
  Chlorhexidine 0.20% mouth rinse, three times daily for 10 days.
- **Analgesics:**
  Non-steroidal anti-inflammatory drugs prescribed as needed.

Following surgical treatment, all patients were enrolled in a supportive peri-implant maintenance program with professional recall visits scheduled at 6-month intervals. During each maintenance appointment, professional supra- and subgingival plaque control was provided, and oral hygiene procedures were reviewed and reinforced. Patients were instructed and re-motivated to maintain adequate home plaque control using a manual or electric toothbrush, together with interdental cleaning aids such as interdental brushes and/or dental floss, according to individual needs.

### 2.5. Outcome Measures

The primary outcomes were changes in probing depth (PD) and radiographic bone level variation between baseline and 6 months. PD was assessed using a standardized periodontal probe at six sites per implant. Standardized periapical radiographs were obtained using the long-cone paralleling technique with individualized silicone bite registration devices to ensure reproducible positioning. Radiographic bone level was assessed as the distance from the implant shoulder to the first bone-to-implant contact on mesial and distal aspects. Calibration was performed using known implant thread pitch or implant dimensions as internal reference markers to correct for potential magnification errors. Probing depth (PD) was measured at six sites per implant using a PCP-UNC 15 periodontal probe (Hu-Friedy, Chicago, IL, USA). A standardized light probing force not exceeding approximately 25–30 g, in accordance with established periodontal guidelines, was applied. All clinical measurements were performed by an experienced and calibrated periodontist. The examiner performing clinical measurements was blinded to treatment allocation during data collection and analysis, as data extraction from clinical records was performed without access to group identifiers. Radiographic bone-level analysis was only performed for implants treated without adjunctive regenerative therapy, whereas clinical outcomes were analyzed in the entire cohort. This approach was adopted to minimize the influence of grafting procedures on radiographic assessment of bone fill while preserving the clinical representativeness of the overall peri-implantitis cohort. Secondary outcomes included implant survival and persistence of bleeding and/or suppuration at follow-up.

### 2.6. Statistical Analysis

All statistical analyses were performed using dedicated statistical software (e.g., SPSS version 29.0, IBM Corp., Armonk, NY, USA or equivalent). The level of statistical significance was set at α = 0.05. Descriptive statistics were calculated for all variables. Continuous variables, including probing depth (PD) and radiographic bone level changes, were expressed as mean ± standard deviation (SD). Categorical variables were reported as frequencies and percentages. Normality of continuous variables was assessed using the Shapiro–Wilk test, given the limited sample size. Since data distribution did not significantly deviate from normality, parametric tests were applied for the main analyses.

Intragroup comparisons between baseline and 6-month follow-up were performed using paired t-tests. Intergroup comparisons between the electrolytic and conventional decontamination groups were performed using independent samples t-tests. The primary outcomes were changes in probing depth (ΔPD) and radiographic bone level variation between baseline and 6 months. These variables were analyzed as continuous outcomes and expressed as mean differences with 95% confidence intervals (CIs). Due to the absence of implant failures during the observation period and the limited number of residual inflammatory events, these variables were analyzed descriptively only. Given that regenerative procedures were performed in defects with a vertical component > 3 mm irrespective of the decontamination protocol, regenerative therapy was considered a potential confounding factor when interpreting radiographic bone fill outcomes. Because of the relatively small sample size and retrospective design, the statistical analysis should be considered exploratory. Effect sizes and confidence intervals are reported alongside *p*-values to improve clinical interpretability.

## 3. Results

**Figure 5:**
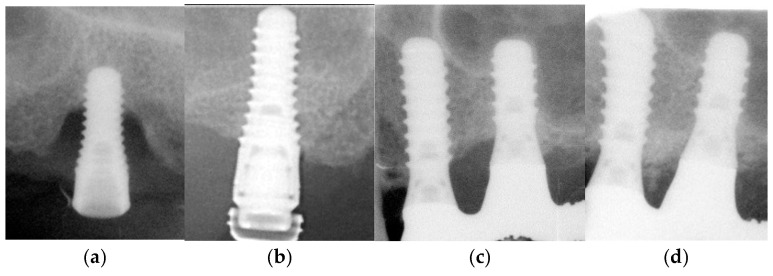
Representative radiographic cases. (**a**) Baseline radiographic bone loss in the control group; (**b**) 6 months of healing after surgical treatment and grafting; (**c**) baseline mesial bone defect in the test group; (**d**) 6 months of healing after electrolytic decontamination and grafting.

A total of 20 implants, all with the same SLA surface (10 per group), were included. Baseline demographic and clinical characteristics are summarized in Table 1. There were no statistically significant differences observed between groups for the available baseline variables. The distribution of regenerative procedures was balanced between groups, with four regenerated defects in each treatment group (Figure 5).

Both treatment protocols resulted in significant clinical and radiographic improvements at 6 months. Radiographic bone-level changes were only analyzed in the non-regenerative cases, excluding the 8 implants that received adjunctive regenerative treatment, in order to avoid potential distortion of bone fill measurements related to grafting procedures. In the remaining non-regenerative cases, radiographic bone levels improved in both groups over the 6-month follow-up. Clinical outcomes, including probing depth reduction, were instead analyzed in the overall cohort, as regenerative treatment represented part of the routine surgical management of peri-implantitis defects according to intraoperative defect characteristics.

Probing depth (PD) improved in both groups. In the electrolytic group, mean PD decreased from 6.7 ± 0.95 mm at baseline to 4.0 ± 0.1 mm at 6 months (ΔPD = −2.7 mm). In the conventional group, PD decreased from 6.8 ± 1.03 mm to 3.3 ± 0.1 mm (ΔPD = −3.5 mm), showing a greater numerical reduction than the electrolytic group.

Radiographic bone-level analysis was restricted to non-regenerative cases only. In these sites, radiographic bone loss improved in both groups over the 6-month follow-up. In the electrolytic group, mean bone loss decreased from 2.89 ± 0.70 mm at baseline to 0.78 ± 0.70 mm at 6 months (ΔBL = 2.11 mm). In the conventional group, bone loss decreased from 2.92 ± 1.83 mm to 0.94 ± 1.14 mm (ΔBL = 1.98 mm). The magnitude of radiographic improvement was therefore comparable between the two decontamination protocols in the non-regenerative subgroup (Table 2 and Table 3).

## 4. Discussion

This retrospective study evaluated the clinical and radiographic outcomes of two implant surface decontamination protocols used during surgical treatment of peri-implantitis: a conventional mechanical approach based on titanium curettes, chitosan brushes, and glycine powder air-polishing, and an electrolytic cleaning protocol.

Within the limitations of the present investigation, both treatment modalities resulted in significant improvements in probing depth reduction and radiographic bone fill after 6 months. The absence of stratification according to defect morphology may also have influenced treatment outcomes.

Moreover, the adjunctive regenerative procedures performed in selected defects may have acted as a confounding factor when interpreting radiographic bone fill outcomes, since regenerative therapy itself can significantly influence the extent of defect resolution and bone gain [11]. However, no statistically significant intergroup differences were observed, suggesting that electrolytic decontamination may represent a clinically effective alternative to conventional implant surface decontamination approaches in the surgical management of peri-implantitis. Regenerative therapy was performed in defects with an intraosseous component greater than 3 mm when the defect morphology was considered suitable for grafting. Although this reflected routine clinical decision-making rather than a study-driven intervention, it may have influenced both probing depth reduction and radiographic bone fill. Therefore, regenerative treatment should be considered a potential residual confounding factor when interpreting the present findings.

The treatment of peri-implantitis remains one of the most challenging conditions in implant dentistry due to the difficulty of achieving predictable implant surface decontamination while preserving the biological characteristics of the titanium surface [31,32]. Conventional mechanical methods, including curettes and air-polishing systems, have demonstrated clinical efficacy, although concerns remain regarding incomplete biofilm removal and potential alterations of implant surface microtopography. In this context, electrolytic cleaning has recently emerged as a promising strategy for removing biofilms and organic contaminants through electrochemical reactions without inducing mechanical damage to the implant surface.

The rationale behind electrolytic decontamination is biologically attractive. By generating hydrogen bubbles at the implant surface, the electrochemical process may facilitate disruption of the bacterial biofilm even within complex implant macrogeometries and roughened surfaces. Furthermore, previous experimental studies suggested that electrolytic cleaning may restore titanium surface wettability and biocompatibility, potentially favoring re-osseointegration of previously contaminated implant surfaces. These theoretical advantages have generated increasing interest in the use of electrolytic systems during peri-implantitis surgery.

In the present study, both groups demonstrated marked reductions in probing depth and substantial radiographic defect fill. The magnitude of PD reduction observed in both groups exceeded thresholds generally considered clinically relevant in peri-implantitis therapy. Similarly, the radiographic bone gain observed after treatment suggests favorable healing of the peri-implant defects irrespective of the decontamination protocol employed.

Interestingly, although the conventional treatment group showed a numerically greater reduction in probing depth, this difference did not reach statistical significance. Several factors may explain this finding. First, the relatively small sample size may have limited the ability to detect modest intergroup differences. Second, peri-implantitis outcomes are known to be multifactorial and influenced not only by implant surface decontamination itself, but also by defect morphology, host response, maintenance compliance, implant design, and prosthetic factors [33]. Finally, the adjunctive regenerative component adopted in defects deeper than 3 mm may have contributed substantially to the overall healing response in both groups, potentially reducing the detectable impact of the decontamination strategy alone [23].

The absence of significant differences between groups should not necessarily be interpreted as a lack of clinical relevance of electrolytic cleaning. On the contrary, demonstrating comparable outcomes to established conventional approaches may itself represent an important finding, particularly considering the theoretical advantages of electrolytic systems in terms of surface preservation and biofilm removal. In this regard, the present results are consistent with previous investigations reporting favorable clinical outcomes following electrolytic implant surface decontamination in peri-implantitis treatment.

Another clinically relevant aspect is that no implant losses were observed during the observation period, indicating that both treatment protocols were associated with satisfactory short-term implant survival. This finding further supports the effectiveness of surgical intervention combined with thorough implant surface decontamination and appropriate defect management.

The present study has several limitations that should be acknowledged. A limitation of the present study is the retrospective design, which resulted in incomplete availability of some clinically relevant baseline variables. In particular, information regarding peri-implant defect morphology, implant loading duration, and oral hygiene-related parameters was not consistently documented in the clinical records and therefore could not be included in the baseline comparison or adjusted statistical analyses. Consequently, the potential influence of these factors on the observed outcomes cannot be fully excluded. The retrospective design inherently limits control over confounding variables and introduces the potential for selection bias. Moreover, the sample size was relatively limited, and therefore the study should be considered exploratory. Furthermore, the follow-up period was restricted to 6 months, preventing assessment of long-term stability. In addition, microbiological and patient-reported outcome measures were not included, which may have provided further insight into the biological and clinical effects of the different decontamination approaches. In addition, detailed information regarding peri-implant defect morphology and graft-related characteristics (e.g., graft volume and configuration) was not consistently available in the clinical records. Therefore, stratified analyses or multivariable adjustment including these variables could not be performed. As a result, residual confounding related to regenerative procedures cannot be completely excluded.

An additional limitation of the present study is that implant design characteristics, including the distinction between bone-level and tissue-level implants, were not consistently available in the retrospective clinical records and therefore could not be systematically analyzed. As implant macrodesign and transmucosal configuration may influence plaque accumulation, accessibility for decontamination, and peri-implant defect morphology, their potential impact on treatment outcomes cannot be fully excluded.

A further methodological consideration concerns the inclusion of regenerative cases within the overall clinical cohort. Regenerative therapy was performed as part of routine surgical management in defects with an intraosseous component and morphology considered suitable for grafting. While these cases were retained in the analysis of clinical outcomes to preserve the representativeness of the treated peri-implantitis population, they were excluded from the radiographic bone-level analysis in order to avoid confounding related to graft-induced defect fill. This distinction should be taken into account when interpreting the present findings.

Despite these limitations, the study also presents several strengths. Clinical and radiographic parameters were collected using standardized protocols, baseline comparability between groups was confirmed, and the study reflects real-world clinical conditions. Future prospective randomized controlled trials with larger samples and longer follow-ups are needed to clarify whether electrolytic cleaning provides additional biological or clinical advantages over established mechanical protocols. Particular attention should be given to re-osseointegration potential, long-term disease recurrence, and patient-centered outcomes.

## 5. Conclusions

Within the limitations of this retrospective study, both electrolytic and conventional mechanical decontamination protocols resulted in significant clinical and radiographic improvements after surgical treatment of peri-implantitis. Although no statistically significant differences were observed between groups, electrolytic cleaning achieved outcomes comparable to conventional therapy and may be considered a viable alternative for implant surface decontamination during peri-implantitis surgery. Further prospective randomized controlled trials with larger samples and longer follow-ups are warranted to clarify the potential biological and clinical advantages of electrolytic decontamination.

## Figures and Tables

**Table 1:** Demographic and Clinical Characteristics of the Study Population.

Variable	Electrolytic (*n* = 10)	Conventional (*n* = 10)	*p*-Value
Age (years), mean ± SD	63.6 ± 9.88	59.9 ± 10.7	0.44
Male/Female	6/4	5/5	1.00
Posterior sites, *n* (%)	9 (90%)	7 (70%)	0.58
Anterior sites, *n* (%)	1 (10%)	3 (30%)	0.58
Baseline PD (mm), mean ± SD	6.7 ± 0.95	6.8 ± 1.03	0.81
Baseline bone loss (mm), mean ± SD	4.88 ± 0.70	4.88 ± 1.83	1.00
Baseline BOP, *n* (%)	7 (70%)	6 (60%)	1.00
Regenerative therapy, *n* (%)	4 (40%)	4 (40%)	1.00

This table summarizes the demographic characteristics of the study population and the distribution of treated sites, including clinical outcomes and types of decontamination procedures performed, according to the available data from the clinical records examined.

**Table 2:** Clinical parameters.

Parameter	Electrolytic (Mean ± SD)	Conventional (Mean ± SD)	*p*-Value
Initial PD (mm)	6.7 ± 0.95	6.8 ± 1.03	0.81
Final PD (mm)	4.0 ± 0.1	3.3 ± 0.1	-
Initial bone loss (mm)	2.89± 0.70	2.92 ± 1.83	1.00
Final bone loss (mm)	0.78 ± 0.70	0.94 ± 1.14	-

Baseline and 6-month clinical parameters. Radiographic bone-level outcomes were only calculated for non-regenerative cases (*n* = 12), after exclusion of implants treated with adjunctive regenerative therapy. Probing depth outcomes refer to the full cohort (*n* = 20).

**Table 3:** Within-group changes and between-group comparison of clinical and radiographic outcomes from baseline to follow-up.

Outcome	Electrolytic Δ (95% CI)	Conventional Δ (95% CI)	Between-Group Difference (95% CI)	*p*-Value
Δ PD (mm)	−2.7 (−3.36 to −2.04)	−3.5 (−4.22 to −2.78)	0.8 (−0.15 to 1.75)	0.09
Δ Radiographic Bone loss (mm) *	−2.11	−1.98	0.13	NR

PD outcomes refer to the full cohort (*n* = 20). * Radiographic bone-level outcomes were only calculated for non-regenerative cases (*n* = 12), after exclusion of the 8 implants treated with adjunctive regenerative therapy, in order to avoid confounding related to graft-induced defect fill. Δ = final value − baseline value; NR = not recalculated/reported. Data are presented as mean change (Δ) with 95% confidence intervals (95% CIs). Negative values for probing depth (PD) indicate a reduction in pocket depth, while positive values for bone loss indicate radiographic bone gain (defect fill) over the observation period. Between-group differences represent the mean difference in changes between the electrolytic and conventional decontamination groups. No statistically significant differences were observed between the two treatment modalities for either probing depth reduction (*p* = 0.09) or radiographic bone gain (*p* = 1.00).

## Data Availability

Data available upon request.
